# Neopterin in patients with COPD, asthma, and ACO: association with endothelial and lung functions

**DOI:** 10.1186/s12931-024-02784-4

**Published:** 2024-04-18

**Authors:** Yangli Liu, Fengjia Chen, Zhimin Zeng, Chengcheng Lei, Dubo Chen, Xiaoyu Zhang

**Affiliations:** 1 Division of Pulmonary and Critical Care Medicine, The First Affiliated Hospital of Sun Yat-sen University, Province Guangdong, Guangzhou, 510080 PR China; 2 Laboratory Medicine, The First Affiliated Hospital of Sun Yat-sen University, Guangzhou, Province Guangdong, 510080 PR China; 3 Department of Hypertension and Vascular Disease, The First Affiliated Hospital of Sun Yat-sen University, Province Guangdong, Guangzhou, 510080 PR China; 4 National-Guangdong Joint Engineering Laboratory for Diagnosis and Treatment of Vascular Diseases, Guangzhou, 510080 PR China; 5 Key Laboratory on Assisted Circulation, Ministry of Health, Guangzhou, 510080 PR China

**Keywords:** Asthma-COPD overlap (ACO), Chronic obstructive pulmonary disease (COPD), Asthma, Neopterin, Endothelial dysfunction

## Abstract

**Background and objective:**

Endothelial dysfunction has been widely recognized in chronic airway diseases, including chronic obstructive pulmonary disease (COPD) and asthma; however, it remains unclear in asthma-COPD overlap (ACO). Neopterin (NP), a metabolite of guanosine triphosphate, is a novel biomarker for identifying the increased risk of adverse cardiovascular events. This study aims to investigate the association of NP with endothelial dysfunction and impaired lung function in COPD, asthma, and ACO patients.

**Methods:**

A total of 77 subjects were prospectively recruited. All the participants underwent lung function test, endothelial function evaluation, including pulse wave velocity (PWV) and flow-mediated dilation (FMD), and blood sample detection. Moreover, the effect of NP on endothelial cells (ECs) in anoxic environments was assessed in vitro.

**Results:**

Endothelial function was significantly decreased in the COPD and ACO patients compared with that in the healthy controls (*P* < 0.05). Forced expiratory volume in 1 s (FEV1) was negatively correlated with PWV and positively correlated with FMD (*P* < 0.05). NP was significantly increased in patients with chronic respiratory diseases compared with that in the control group, with COPD being the highest, followed by asthma, and ACO as the last (*P* < 0.05). The plasma level of NP exhibited negative correlations with FEV1 and positive correlations with PWV (*P <* 0.05). In vitro, a high level of NP increased the reactive oxygen species (ROS) and decreased the mitochondrial membrane potential (ΔΨm) of ECs dose-dependently in a hypoxic environment (*P* < 0.05).

**Conclusion:**

NP was related to disease severity of chronic airway diseases and involved in the pathogenesis of endothelial dysfunction. A high NP level may contribute to endothelial dysfunction by increasing the oxidative stress of ECs dose-dependently in a hypoxic environment. Our findings may provide a novel evaluation and therapeutic target for endothelial dysfunction related to chronic airway diseases.

## Introduction

Asthma and chronic obstructive pulmonary disease (COPD) are two of the most prevalent chronic airway diseases that pose a major public health problem [1, 2]. Patients with features of asthma and COPD, defined as asthma-COPD overlap (ACO), have been reported to have more rapid disease progression and higher mortality [3]. Accumulating evidence indicates that asthma, COPD, and ACO are correlated with higher risk of cardiovascular events [4–6]. The formation of local and systemic chronic inflammation [7], activation of cytokines and phlogogenic mediators, and involvement of oxidative stress [8] may lead to impairment in endothelial function, which is the link between chronic airway diseases and cardiovascular risk.

Given the inflammatory response to vascular injury, endothelial dysfunction has been widely recognized in COPD [4] and asthma [9] patients with higher cardiovascular risk and associated with pulmonary disease severity. Endothelial inflammation stimulates the production of proinflammatory cytokines, such as interleukin (IL)-6, IL-17, and IL-10 in endothelial cells (ECs) [10–12]. Despite the available data on the changes of cytokine levels and the bioassay of endothelial function, including pulse wave velocity (PWV) and flow-mediated dilation (FMD), in patients with COPD and asthma [9, 13], evidence for their participation in the development of systemic inflammation and endothelial function in ACO patients remains minimal.

Neopterin (NP), a metabolite of guanosine triphosphate, is produced by activated macrophages after their stimulation by γ-interferon [14]. NP has been extensively used as a novel biomarker of immune activation during inflammation and stress. Previous studies have reported that patients with coronary artery disease or hypertension have elevated plasma concentration of NP, which is associated with endothelial dysfunction [15]. However, the effect of NP on endothelial function and its association with lung function in COPD, asthma, and ACO patients have not yet been validated.

Accordingly, in the current study, we investigated the relationships among inflammatory cytokines, biomarker NP level, endothelial function (PWV and FMD), and lung function in COPD, asthma, and ACO patients, and explored the underlying mechanisms by detecting endothelial reactive oxygen species (ROS) and mitochondrial membrane potential (ΔΨm) after stimulating ECs with different concentrations of NP. This study may provide valuable knowledge for future clinical studies that focus on a useful biomarker to detect endothelial dysfunction and impaired lung function in patients with chronic airway diseases.

## Methods

### Subjects

This study was approved by the Human Experimentation and Ethics Committees of Sun Yat-sen University (No. [2021]072), and all the subjects signed a written informed consent. A total of 77 subjects were prospectively included from the division of pulmonary and critical care medicine of the First Affiliated Hospital of Sun Yat-sen University. The diagnosis of asthma and COPD was based on the Global Initiative for Asthma and the Global Initiative for Chronic Obstructive Lung Disease guidelines [1, 16]. The diagnosis of ACO was based on the following conditions: (1) asthma patients aged 40 years or above with persistent airflow restriction [post-bronchodilator forced expiratory volume in 1 s (FEV1) and forced vital capacity (FVC) ratio < 0.7] and had smoke or significant biomass exposure (≥ 10 pack-years for smoke and ≥ 100 h/year for wood or coal), and (2) COPD patients with a history of asthma before the age of 40 years or present a positive bronchodilator response test. A total of 17 control subjects with normal lung function (FEV1 ≥ 80% predicted and FEV1/FVC ≥ 0.7) were recruited from healthy nonsmokers. Subject exclusion criteria include the following: (1) patients with other coexisting pulmonary disease, such as interstitial lung disease, pulmonary embolism, bronchiectasis, or lung cancer; (2) patients with acute respiratory tract infection, surgery, or acute exacerbation within 1 month; and (3) patients with chronic cardiovascular diseases (CVDs), metabolic disorders, and renal or liver dysfunctions.

### Lung function test

All the subjects underwent lung function test following the recommendations of the American Thoracic Society and the European Respiratory Society. All the subjects underwent lung function test in a reproducible manner, and the best values were selected. The parameters, which included FVC, FEV1, percent predicted values of these parameters (%FVC and %FEV1), FEV1/FVC ratio, and maximum voluntary ventilation (MVV), were collected. Bronchodilator response test was performed 15–20 min after the inhalation of 400 µg of salbutamol. A positive response was defined as a ≥ 12% improvement in %FEV1 and an absolute increase of ≥ 200 mL.

### Endothelial function evaluation

FMD was assessed to evaluate endothelial function by using high-resolution ultrasonography (UNEX EF 38G, OMRON, Japan) in accordance with the current guidelines [17]. In summary, the resting diameter and flow data of the right brachial artery were measured. Then, supra-systolic forearm occlusion (50 mmHg above systolic blood pressure) was performed for 5 min, the cuff was subsequently deflated, and vascular dilation response was measured. FMD was calculated as the maximum percentage of change from the resting diameter to the peak diameter of the brachial artery after cuff deflation. A decrease in FMD indicates an increase in cardiovascular risk.

### Arterial stiffness

Arterial stiffness was assessed via brachial-ankle PWV with applanation tonometry (BP-203RPE III, OMRON, Japan) by following the current guidelines [18]. A plethysmographic sensor was attached to the cuff wrapped around the brachial arm and ankles and used to record pulse pressure waveforms simultaneously. The surface distance between the two recording sites (Δd) and transit time (Δt) were recorded. PWV was calculated as PWV = Δd/ΔT (cm/s). The mean value of the left and right PWV was used for data analysis. An increase in PWV indicates greater arterial stiffness and cardiovascular risk.

### Blood sample collection and measurement of inflammatory markers

Venous blood was collected from all the subjects after fasting overnight for at least 8 h. Inflammatory markers, i.e., serum IL-10, IL-17, IL-6, and NP, were measured using commercial enzyme-linked immunosorbent assay (MEIMIAN, Wuhan, China).

### Cell cultures

Human aortic ECs (HAECs) were cultured in endothelial growth medium (EGM)-2 (Lonza, Verviers, Belgium) and exposed to hypoxia (1% oxygen) as optimized and characterized in previous studies.

### ROS assay

Intracellular ROS levels were measured using the oxidant-sensing probe dichlorodihydrofluorescein diacetate (DCFH-DA). HAECs grown in six-well culture plates were exposed to hypoxia with or without NP treatment. After 24 h, the cells were incubated with 10 µM of DCFH-DA diluted in EGM-2 (serum-free) at 37 ℃ in the dark. After 20 min of incubation, the cells were washed three times with phosphate-buffered saline (PBS) and determined under flow cytometry (BD Biosciences, CA, USA).

### ΔΨm measurement

ΔΨm was measured using JC-1 dye (Beyotime) in accordance with the manufacturer’s instructions. HAECs were cultured in six-well culture plates and exposed to hypoxia with or without NP treatment for 24 h. Thereafter, the cells were dyed with JC-1 staining solution at 37 ℃ for 20 min and observed under a fluorescence microscope (Leica, Wetzlar, Germany). Red fluorescent JC-1 aggregates formed in the hyperpolarized membranes, while green fluorescent JC-1 monomers indicated membrane depolarization. The higher the ratio of red-to-green fluorescence, the more intact the mitochondrial membrane.

### Statistical analysis

SPSS 25.0 (SPSS, Chicago, IL) was used for statistical analysis. Normally distributed data were expressed as mean ± SD and non-normally distributed data as median (percentile 25–75). The comparison of differences among the four groups was conducted via ANOVA with post-hoc contrast tests by using the least significant difference test. Categorical variables were compared using the chi-squared test. Potential correlations among variables were explored using Pearson’s correlation test. Statistical significance was considered when *P* < 0.05.

## Results

### Clinical characteristics

The clinical characteristics of all the subjects are provided in Table 1. Among the 77 subjects, 20 were diagnosed with asthma, 20 were ACO patients, 20 had COPD, and 17 were healthy controls. No significant difference in age, height, weight, and blood pressure was found among the four groups. Patients in the COPD group were all males with lower body mass index (BMI) compared with the subjects in the other groups. The three patient groups exhibited lower lung function than the healthy controls. In addition, the spirometry parameters, including FVC% predicted, FEV1% predicted, the FEV1/FVC ratio, and MVV% predicted, in the ACO group were between those in the asthma and COPD groups.

**Table 1:** Basic characteristics of the subjects

	Asthma (*n* = 20)	ACO (*n* = 20)	COPD (*n* = 20)	HC (*n* = 17)	*P* value
Age	57.2 ± 9.3	56.8 ± 9.0	58.9 ± 6.6	52.5 ± 6.9	0.113
Male, n (%)	12 ((60)	16 (80)	20 (100)	12 (71)	0.019
Height, m	1.6 ± 0.1	1.7 ± 0.1	1.7 ± 0.1	1.6 ± 0.1	0.121
Weight, kg	63.8 ± 9.0	65.1 ± 11.8	58.6 ± 8.8	63.8 ± 8.2	0.152
BMI, kg/m^2^	23.6 ± 2.8	23.2 ± 3.2	20.7 ± 3.1	24.0 ± 2.9	0.006
SBP, mmHg	131 ± 18	131 ± 16	131 ± 15	125 ± 17	0.681
DBP, mmHg	80 ± 9	81 ± 11	81 ± 9	76 ± 12	0.333
Spirometry					
FVC%pred	94.3 ± 21.2	75.3 ± 16.9	64.1 ± 16.2	105.2 ± 13.2	<0.0001
FEV1%pred	81.9 ± 21.3	48.1 ± 16.8	42.5 ± 18.2	106.8 ± 10.3	<0.0001
FEV1/FVC	70.3 ± 7.5	50.7 ± 8.1	51.7 ± 12.7	83.1 ± 4.8	<0.0001
MVV%pred	80.0 ± 18.6	48.1 ± 20.7	40.5 ± 19.2	99.4 ± 13.2	<0.0001
Vascular evaluation					
PWV, cm/s	1657.2 ± 395.6	1638.4 ± 328.8	1830.3 ± 554.6	1432.4 ± 286.4	0.040
FMD (% )	7.09 ± 3.30	5.36 ± 1.61	5.45 ± 2.71	7.24 ± 3.30	0.063
Inflammatory markers					
IL-10, pg/ml	448.4 (383.4-643.1)	438.8 (368.1-553.1)	462.7 (395.5-530.5)	500.5 (383.7–543.0)	0.472
IL-17, pg/ml	33.4 (5.9–58.6)	11.1 (0.0-37.1)	11.0 (0.9–17.7)	17.5 (8.1–23.4)	0.279
IL-6, pg/ml	27.5 (18.9–37.5)	30.9 (18.5–39.1)	28.2 (18.0-37.9)	32.7 (21.6–56.8)	0.472
NP, nmol/l	8.1 ± 2.6	7.8 ± 2.6	9.0 ± 2.6	5.9 ± 2.7	0.006

Data are shown as mean ± SD, median (interquartile range [IQR]) or n (%)

COPD: chronic obstructive pulmonary disease; ACO: asthma-COPD overlap; HC: healthy control; BMI: body mass index; SBP: systolic blood pressure; DBP; diastole blood pressure; FVC: forced vital capacity; FEV1: forced expiratory volume in one second; MVV: maximal voluntary ventilation; PWV: pulse wave velocity; FMD: flow-mediated dilation; IL; interleukin; NP; neopterin

**Fig. 1:**
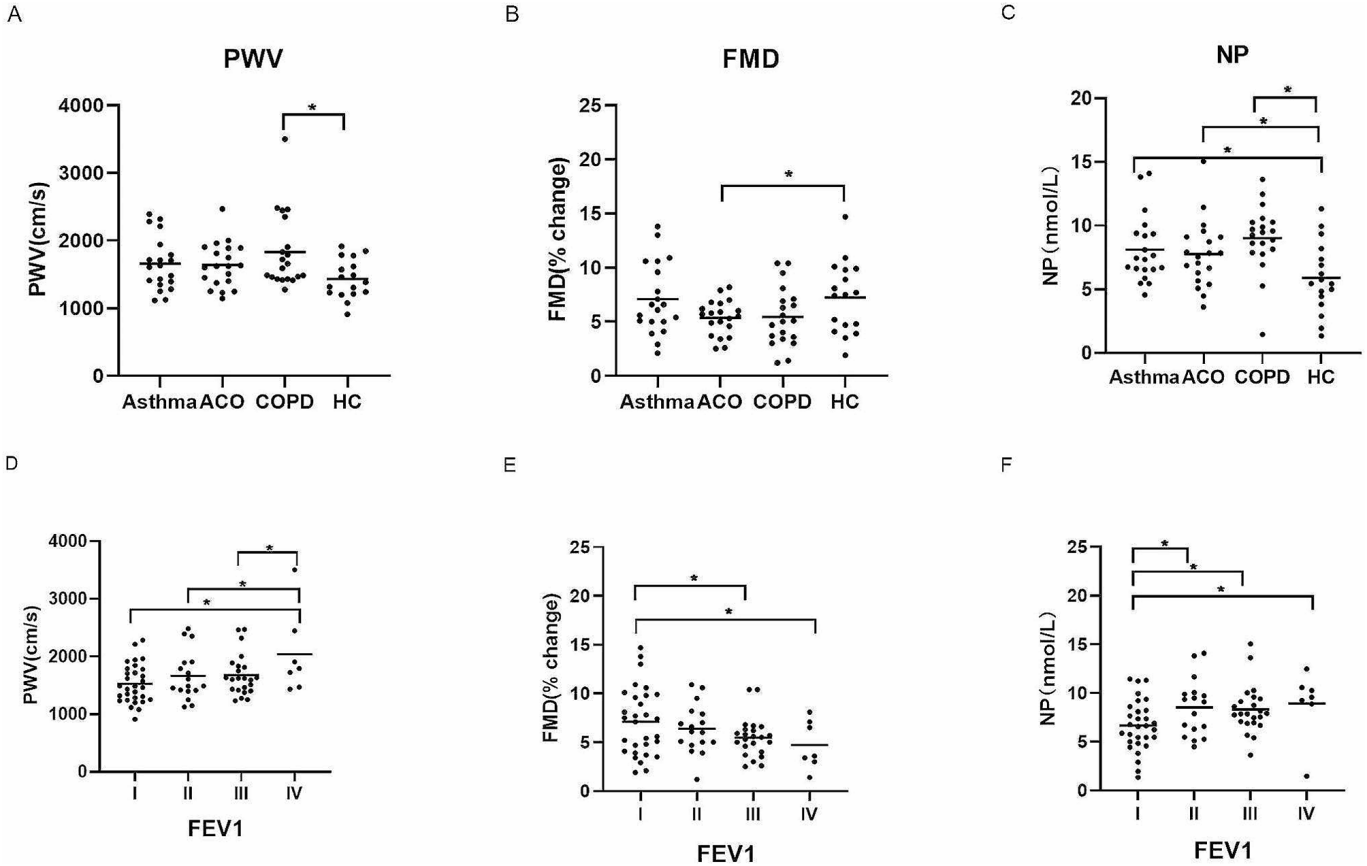
Comparisons of PWV, FMD, and NP in different subgroups. (**A**–**C**): Comparisons of PWV, FMD, and NP in patients with asthma, ACO, COPD, and healthy controls. (**D**–**E**): Comparisons of PWV, FMD, and NP in patients with different lung functions divided by FEV1. (*P < 0.05)

Endothelial function was significantly decreased in the COPD (PWV: 1830.3 ± 554.6 cm/s versus 1432.4 ± 286.4 cm/s, *P* < 0.05) and ACO patients (FMD: 5.36 ± 1.61% versus 7.24 ± 3.30%) as compared to health control (*P* < 0.05). No significant difference in the levels of endothelial function was found among the asthma, ACO, and COPD patients (Fig. 1A and B).

The inflammatory marker NP was significantly increased in patients with chronic respiratory diseases than in the control group, with COPD being the highest, asthma as the second, and ACO as the last. (NP: 9.0 ± 2.6 nmol/L in COPD, 8.1 ± 2.6 nmol/L in asthma, and 7.8 ± 2.6 nmol/L in ACO versus 5.9 ± 2.7 nmol/L in the healthy controls, *P* < 0.05), as shown in Fig. 1C. Other inflammatory markers, including IL-10, IL-17, and IL-6, were comparable in all four groups.

### Comparison among subjects with different lung functions

In accordance with the lung function parameter FEV1, the subjects were further divided into four groups as presented in Table 2: Group I (FEV1 ≥ 80% predicted), Group II (80% > FEV1 ≥ 50% predicted), Group III (50% > FEV1 ≥ 30% predicted) and Group IV (FEV1 < 30% predicted). We then compared clinical manifestation among the four groups. No significant difference was found with regard to age, gender, height, weight, and blood pressure. The subjects with poor lung function had lower BMI, indicating poorer nutrition condition.

**Table 2:** Comparison between different lung function

	I (*n* = 30)	I(*n* = 17)	III (*n* = 23)	IV*n* = 7)	*P * *value*
Age	55.4 ± 8.9	55.1 ± 6.6	58.0 ± 9.3	59.4 ± 3.6	0.445
Male	20 (67)	14 (82)	19 (83)	7 (100)	0.197
Height, m	1.6 ± 0.1	1.7 ± 0.1	1.7 ± 0.1	1.7 ± 0.1	0.057
Weight, kg	64.0 ± 8.5	62.5 ± 6.8	62.9 ± 11.9	57.7 ± 13.1	0.500
BMI, kg/m^2^	24.0 ± 2.7	22.6 ± 2.6	22.3 ± 3.4	20.2 ± 4.5	0.022
SBP, mmHg	126 ± 17	128 ± 18	134 ± 13	131 ± 17	0.357
DBP, mmHg	77 ± 10	79 ± 10	83 ± 11	82 ± 11	0.178
Vascular evaluation					
PWV, cm/s	1524.5 ± 337.3	1664.6 ± 421.2	1667.0 ± 355.7	2037.7 ± 727.6	0.032
FMD (% )	7.1 ± 3.5	6.4 ± 2.5	5.5 ± 2.0	4.7 ± 2.5	0.095
Inflammatory markers					
IL-10, pg/ml	440.8 (382.4-556.4)	445.7 (362.3-598.5)	460.1 (387.0-571.1)	456.2 (448.3-535.8)	0.847
IL-17, pg/ml	21.1 (11.8–35.4)	4.3 (0.0-51.8)	12.9 (3.1–44.0)	12.0 (6.4–16.0)	0.497
IL-6, pg/ml	30.7 (20.5–56.1)	29.2 (18.9–36.2)	23.1 (18.4–38.3)	34.1 (19.4–67.3)	0.701
NP, nmol/l	6.7 ± 2.6	8.5 ± 2.9	8.3 ± 2.4	8.9 ± 3.5	0.043

Data are shown as mean ± SD, median (interquartile range [IQR]) or n (%)

BMI: body mass index; SBP: systolic blood pressure; DBP; diastole blood pressure; PWV: pulse wave velocity; FMD: flow-mediated dilation; IL: interleukin; NP: neopterin

PWV in Group IV was significantly increased compared with those in the three other groups (PWV: 2037.7 ± 727.6 cm/s in Group IV versus 1524.5 ± 337.3 cm/s in Group I, 1664.6 ± 421.2 cm/s in Group II, and 1667.0 ± 355.7 cm/s in Group III, *P*<0.05). FMD was significantly decreased in Groups III and IV compared with that in Group I (FMD: 5.5 ± 2.0% in Group III and 4.7 ± 2.5% in Group IV versus 7.1 ± 3.5% in Group I, *P* < 0.05) (Fig. 1D and E).

With regard to the plasma level of NP, all the patients with impaired lung function exhibited a significant increased level compared with the subjects in Group I (NP: 8.5 ± 2.9 nmol/L in Group II, 8.3 ± 2.4 nmol/L in Group III, and 8.9 ± 3.5 nmol/L in Group IV versus 6.7 ± 2.6 nmol/L in Group I, *P* < 0.05). No significant difference in IL-10, IL-17, and IL-6 was found among the four groups (Fig. 1F).

### Relationships between lung function and endothelial parameter

**Fig. 2:**
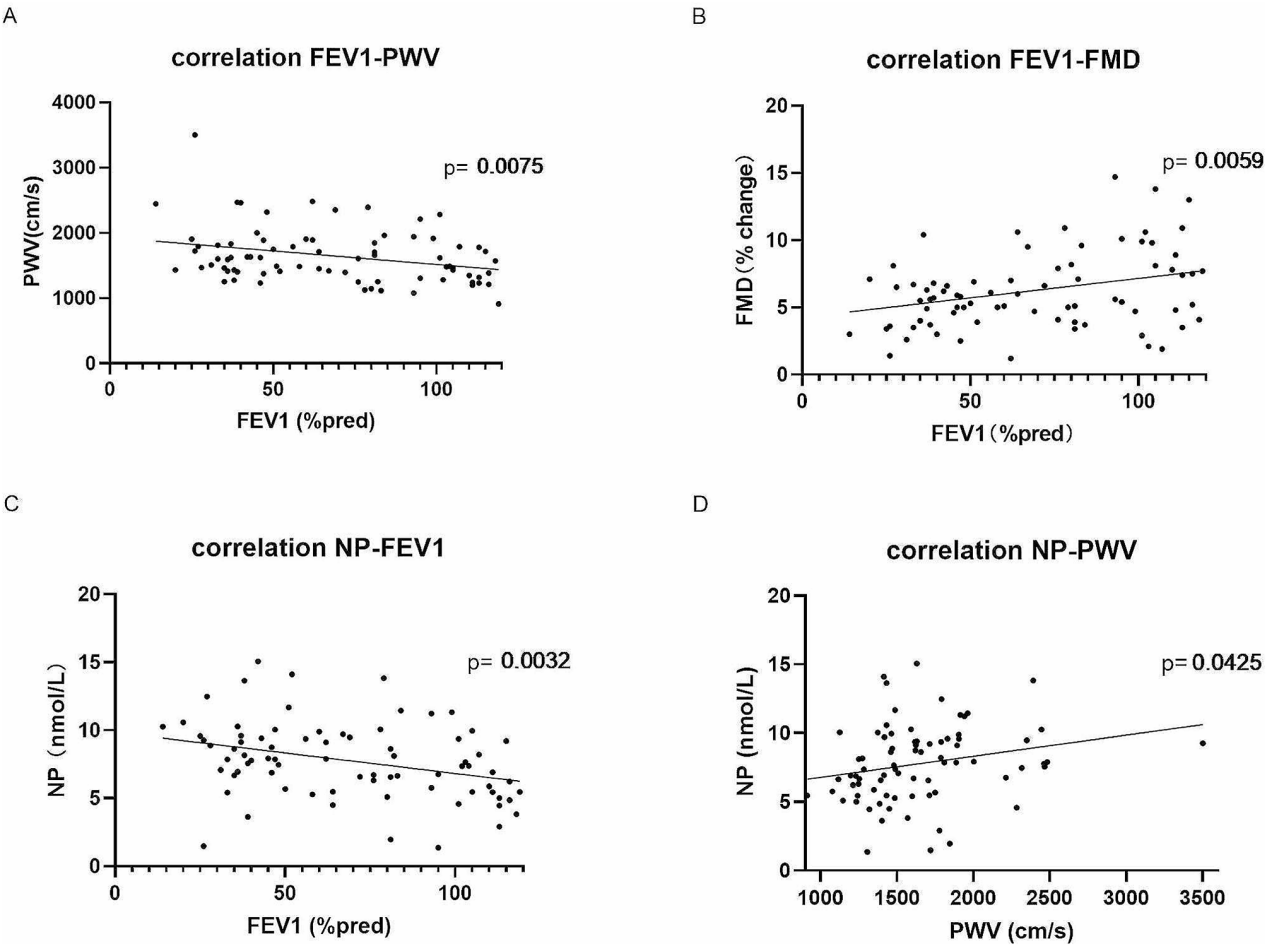
Correlation among lung function, endothelial function, and NP. (**A**): Correlation between FEV1 and PWV. (**B**): Correlation between FEV1 and FMD. (**C**): Correlation between NP and FEV1. (**D**): Correlation between NP and PWV. (*P < 0.05)

All the study subjects were aggregated as a group to explore the possible correlations between lung function and endothelial parameter. The lung function FEV1 was found to be negatively correlated with PWV but positively correlated with FMD (*P* < 0.05) (Fig. 2A and B). The plasma level of NP exhibited negative correlations with FEV1 and positive correlations with PWV (*P* < 0.05) (Fig. 2C and D).

### Effects of NP on oxidative stress in HAECs exposed to hypoxia

**Fig. 3:**
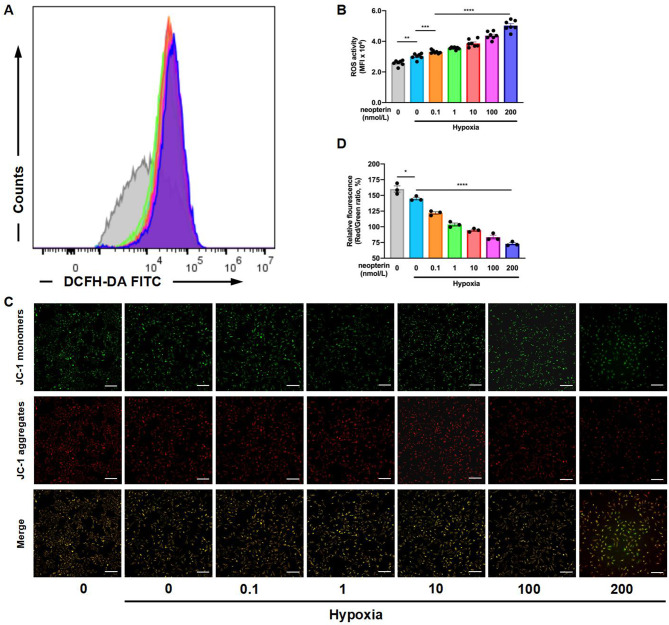
Effects of NP on oxidative stress in HAECs exposed to hypoxia. (**A**–**B**): ROS accumulation in HAECs with or without neopterin treatment (0-200 nmol /L) were detected using DCFH-DA staining in hypoxia environment (*n* = 7). (**C**–**D**): Mitochondrial function in HAECs with or without NP treatment (0–200 nmol /L) was detected through ΔΨm (J-aggregates/monomer ratio) in a hypoxic environment (*n* = 3). (*P < 0.05, ****P < 0.0001)

The effects of NP on the hypoxia-mediated accumulation of ROS in HAECs at 24 h were investigated using DCFH-DA staining. The flow cytometry results showed that the generation of ROS triggered by hypoxia in HAECs was intensified by NP treatment dose-dependently (Fig. 3A and B). Then, we assessed the effects of NP on mitochondrial function through ΔΨm detection in HAECs exposed to hypoxia. As shown in Fig. 3 C and 3D, ΔΨm damage was measured using JC-1 dye. Reduced ΔΨm (J-aggregates/monomer ratio was decreased) was detected in a hypoxia environment and was worsen with the treatment of NP dose-dependently compared with the group without NP treatment (*P* < 0.0001). These data suggest that NP treatment affected oxidative stress dose-dependently in hypoxic salvage.

## Discussion

The findings of the present study revealed a significant correlation between lung function measured by FEV1 and vascular endothelial dysfunction by using PWV and FMD in patients with COPD, asthma, and ACO. Moreover, reduced FEV1, increased PWV, and decreased FMD were significantly associated with elevated NP level in patients with varying degrees of disease severity, providing a useful biomarker for the early detection of endothelial dysfunction. In addition, a high NP level increased the oxidative stress of ECs dose-dependently in a hypoxic environment, and this phenomenon can be correlated with inflammatory vascular injury. This study demonstrates the association among NP, lung function, and endothelial dysfunction in patients with chronic airway diseases for the first time, suggesting that serum NP may be attributed to cardiovascular risk as a biomarker of systemic inflammation and a participant of endothelial dysfunction in obstructive airway diseases.

Chronic airway diseases, including asthma, COPD, and ACO, have been reported to be associated with the risk of CVD [4]. The early identification of changes in the cardiovascular system before the onset of CVD is of considerable significance for preventing such diseases. Endothelial dysfunction occurs prior to the formation of atherosclerotic plaque and interferes with the normal homeostasis of vascular integrity, which can be detected early with the noninvasive measurement of FMD and PWV [19]. Chronic airway diseases have been reported to present with endothelial dysfunction by several studies [9, 13] and our previous studies [20, 21]. Individuals with COPD [13] and asthma [9] have exhibited vascular impairments, resulting in increased vascular stiffness (PWV) and worse endothelial function (FMD) in systemic arteries. In the current study, we extended these observations in asthma and COPD and found that the endothelial dysfunction of ACO was an intermediate condition between those of asthma and COPD. Moreover, a positive correlation existed between lung function assessed via FEV1 and endothelial function, suggesting that lung function is more important than disease type in predicting endothelial dysfunction. These findings underscore the importance of the early detection of vascular dysfunction in patients with chronic airway diseases accompanied by decreased lung function.

Inflammation plays an important role in the occurrence and progression of atherosclerosis and cardiovascular events. NP is considered a biomarker of inflammation and immune system activation. Elevated NP level has been reported to identify subjects at higher risk for major adverse cardiovascular events [22]. Previous investigations have also shown a close relationship between NP level and arterial stiffness and endothelial function in patients with hypertension [15]. In line with the preceding evidence, we first reported that NP level was higher in asthma, COPD, and ACO patients than in the healthy controls and significantly associated with PWV and FMD. In addition, we found that NP level was directly related to lung function, implying that NP may be a key factor that links impaired lung function and endothelial dysfunction. Therefore, patients with chronic airway diseases with high NP level may require aggressive CVD risk factor assessment and more intensive therapy. Moreover, NP level may serve as a therapeutic target, because a previous study in hypertension suggested that NP level can be reduced by antihypertensive treatment and the decrease in NP is linearly correlated with the improvement in vascular endothelial function [15]. Thus, more intervention studies that target NP are warranted in patients with obstructive airway diseases to attenuate CVD risk.

Serum NP has been associated with chronic airway obstruction and endothelial dysfunction; however, the mechanism remains unclear and probably multifactorial, involving hypoxia, inflammation, and increased ROS. Our previous studies have demonstrated that ROS and mitochondrial function are involved in the modulation of the endothelial function under various physiologic and pathologic conditions [23, 24]. In addition, the amount of NP has been reported to be related to its ability to release ROS and the toxic effects caused by enhancing ROS [25]. Therefore, NP can be regarded as an indirect estimate of oxidative stress that emerges during cell-mediated immune response. Here, we detected the ROS and mitochondrial function of ECs in vitro to determine whether NP was related to cell function and found that a high NP level could increase ROS and reduce ΔΨm in an anoxic environment dose-dependently. This finding is consistent with the effect of other inflammatory cytokines on oxidative stress [26]. Thus, the increased level of ROS and decreased ΔΨm induced by elevated NP concentrations may contribute to endothelial dysfunction in chronic airway diseases. The data reported here for the first time proved the association between NP and the development of endothelial dysfunction through the regulation of mitochondrial dysfunction in anoxic environments. Therefore, our current study confirms the importance of NP and oxidative stress in the vascular health of patients with chronic airway diseases, providing a new target for the treatment of airway disease-related cardiovascular comorbidity.

Our study has several limitations. First, the cross-sectional design and the lack of follow-up data limit the analysis of the causal relationship among chronic airway diseases, endothelial dysfunction, serum NP, and future cardiovascular events, warranting further investigation. Second, the sample size of the study is relatively small, which may lead to the trend of some data changes, but without statistical difference. Therefore, studies with a larger sample size are necessary to confirm our conclusions. Third, patients with COPD and poor lung function were all males and presented with significantly decreased BMI, which may underestimate the difference in endothelial function compared with those in the other groups. Finally, although our data suggest that NP may be involved in vascular injury by increasing endothelial cell ROS and decreasing ΔΨm in a hypoxic environment, the specific regulatory mechanisms remain to be explored further.

## Conclusion

In summary, patients with chronic airway diseases, including asthma, COPD, and ACO, with impaired lung function suffer from endothelial dysfunction assessed via PWV and FMD, which are early warnings of cardiovascular risk. As a biomarker of inflammation, NP is related to disease severity and involved in the pathogenesis of endothelial dysfunction. NP may be involved by increasing the oxidative stress of ECs in a hypoxic environment, providing a novel biomarker and therapeutic target for the treatment of chronic airway diseases related to endothelial dysfunction.

## Data Availability

The datasets used during the current study are available from the corresponding author on reasonable request.

## Abbreviations

COPD: chronic obstructive pulmonary disease
ACO: asthma-COPD overlap
NP: neopterin
baPWV: brankial-ankle pulse wave velocity
FMD: flow-mediated dilation
ECs: endothelial cells
IL: interleukin
FVC: forced vital capacity
FEV1: forced expiratory volume in one second
MVV: maximal voluntary ventilation
BMI: body mass index
SBP: systolic blood pressure
DBP: diastole blood pressure
ROS: reactive oxygen species
CVD: cardiovascular disease
